# Bullous pemphigoid mimicking toxic epidermal necrolysis^☆^

**DOI:** 10.1016/j.abd.2024.03.009

**Published:** 2024-11-08

**Authors:** Hiram Larangeira de Almeida Jr., Rodrigo Piltcher da Silva, Valéria Magalhães Jorge

**Affiliations:** a Postgraduate Program in Health and Behavior, Universidade Católica de Pelotas, Pelotas, RS, Brazil; b Department of Dermatology, Universidade Federal de Pelotas, Pelotas, RS, Brazil; c Postgraduate Program in Medicine: Surgical Sciences, Universidade Federal do Rio Grande do Sul, Porto Alegre, RS, Brazil; d Department of General Surgery, Universidade Federal de Pelotas, Pelotas, RS, Brazil

Dear Editor,

Bullous pemphigoid (BP) is a well-known condition triggered by autoantibodies directed against hemidesmosomal proteins involved in the adhesion of basal keratinocytes to the basement membrane. Diagnosis is established by detection of subepidermal bullae with an eosinophil-rich infiltrate on histopathology and the identification of antibodies against the basement membrane zone (BMZ) using direct and or indirect immunofluorescence.^1^

In addition to the classic presentation with disseminated tense bullae, numerous variants of BP are recognized, such as prurigoid, erythrodermic, urticarial, and also those mimicking toxic epidermal necrolysis (TEN)^1^; the urticarial form is the most frequent among the non-bullous forms.^2^

A 52-year-old black female patient with no comorbidities one week after using prednisone, ceftriaxone and pantoprazole for pharyngitis, had presented diffuse erythema with desquamation all over the skin (Fig. 1A). The clinical hypothesis of a drug-related eruption was raised. Given the dissemination and intensity of the condition, methylprednisolone 500 mg IV was administered for five days. After the intravenous corticosteroid, the patient was maintained on daily oral prednisone 60 mg, when small bullae started to appear (Fig. 1B), without mucosal involvement and with mild eosinophilia. On the tenth day, epidermal detachment began to occur, resembling TEN (Fig. 1C and D), there was an increase in the eosinophilia (reaching 3,700 eosinophils/mm^3^.

**Figure 1:**
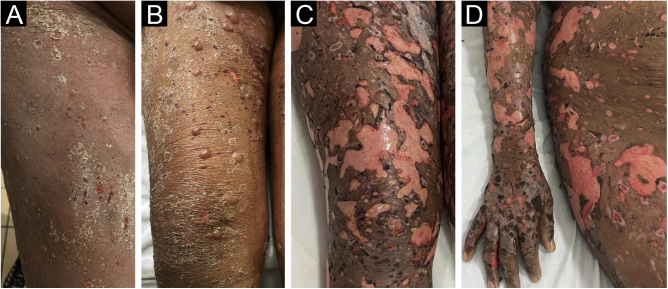
(A) Diffuse erythema and desquamation at disease onset. (B) Reduction in erythema with some bullae. (C and D) Exfoliation of large areas resembling TEN.

As the patient developed fever, intravenous ciprofloxacin was started and prednisone was reduced to 40 mg, which led to the appearance of some new bullae. Due to the risk of a new infection, the dose was reduced to 30 mg, but with the appearance of new bullae, the 40 mg dose was restarted. A bulla was then biopsied and on histopathology subepidermal cleavage, without epithelial necrosis and with significant eosinophilic infiltration in the dermis and inside the bulla were observed (Fig. 2). The immunohistochemical analysis showed IgG deposition on the floor and roof of the bulla (Fig. 3). Indirect immunofluorescence showed linear positivity for IgG in the BMZ. Over the next ten days, the skin exfoliation worsened. On the twentieth day, there were areas of re-epithelialization, erosions of the initial bullaes and new bullae concomitantly (Fig. 4A). The condition was controlled after four weeks of 40 mg prednisone (Fig. 4B). Eosinophilia persists and the patient reports significant pruritus, even with the lesions under control.

**Figure 2:**
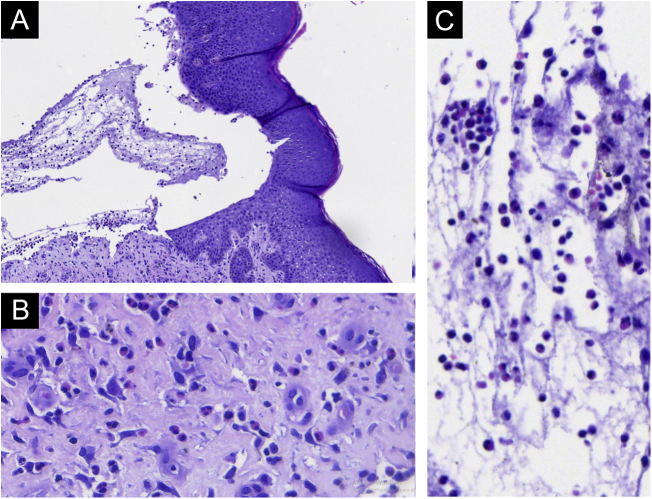
Light Microscopy - (A) Subepidermal bulla, without epithelial necrosis. (B) Eosinophils in the dermis. (C) Eosinophils within the bulla. (Hematoxylin & eosin, × 200 and × 400).

**Figure 3:**
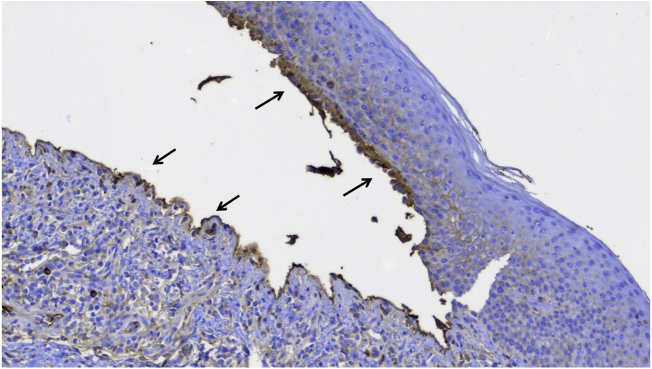
Immunohistochemistry with anti-IgG antibody showing positivity on the floor and roof of the bulla (arrows).

**Figure 4:**
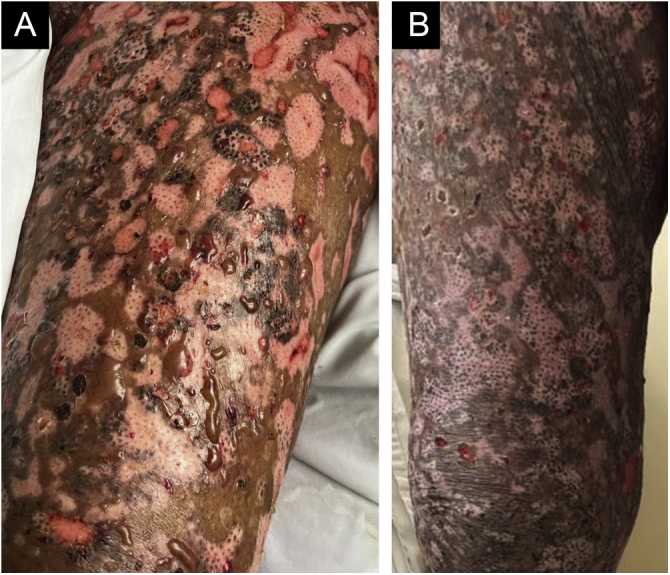
(A) Re-epithelialized hypochromic areas, erosions resulting from the first worsening episode and new bullae. (B) Almost complete re-epithelialization of the lesions.

Mimicking TEN by bullous pemphigoid is known to occur but rare,^3^, ^4^ and the diagnosis can be difficult in the early phase of the disease.^5^ There is also a report of a case triggered by immunotherapy for solid neoplasia.^6^ It is likely that the intense production of antibodies against hemidesmosomal proteins leads to epidermal detachment but without epithelial necrosis as in TEN. Other autoimmune diseases that affect the dermo-epidermal junction can also simulate TEN.^7^

Absence of mucosal involvement may suggest TEN. Peripheral eosinophilia, as in the present case, occurs in 50% of the cases^8^ of BP, with tissue eosinophilia being a criterion for the diagnosis, correlating with the severity of the condition.^9^

The present case documents a rare variant of BP, as well as the initial diagnostic difficulty.

## Financial support

None declared.

## Authors’ contributions

Hiram Almeida Jr.: Approval of the final version of the manuscript; design and planning of the study; drafting and editing of the manuscript; collection, analysis and interpretation of data; effective participation in research orientation; critical review of the manuscript.

Rodrigo Piltcher da Silva: Approval of the final version of the manuscript; design and planning of the study; drafting and editing of the manuscript; collection, analysis and interpretation of data; critical review of the manuscript.

Valéria Jorge: Approval of the final version of the manuscript; design and planning of the study; drafting and editing of the manuscript; collection, analysis and interpretation of data; critical review of the manuscript.

## Conflicts of interest

None declared.
